# The quest for the reliability of machine learning models in binary classification on tabular data

**DOI:** 10.1038/s41598-023-45876-9

**Published:** 2023-10-27

**Authors:** Vitor Cirilo Araujo Santos, Lucas Cardoso, Ronnie Alves

**Affiliations:** 1 Federal University of Pará, PPGCC, Belém, 66075-110 Brazil; 2 Vale Institute of Technology, Belém, 66055-090 Brazil

**Keywords:** Computational science, Computer science, Software

## Abstract

In this paper we explore the reliability of contexts of machine learning (ML) models. There are several evaluation procedures commonly used to validate a model (precision, F1 Score and others); However, these procedures are not linked to the evaluation of learning itself, but only to the number of correct answers presented by the model. This characteristic makes it impossible to assess whether a model was able to learn through elements that make sense of the context in which it is inserted. Therefore, the model could achieves good results in the training stage but poor results when the model needs to be generalized. When there are many different models that achieve similar performance, the model that presented the highest number of hits in training does not mean that this model is the best. Therefore, we created a methodology based on Item Response Theory that allows us to identify whether an ML context is unreliable, providing an extra and different validation for ML models.

## Introduction

Standard Machine Learning (ML) pipelines are built around a training task, which is characterized by a model specification, a training dataset, and an independent, identically distributed evaluation procedure (IID)^1^. The IID evaluation procedure validates the expected predictive performance of a predictor on the data extracted from the training distribution. The problem is that the commonly used evaluation procedures are not tied to specific inductive biases encoded by the trained model; thus, the evaluation procedures do not analyze whether the models learned correctly. So while these types of assessments have contributed to transformational progress in many problem areas, their blind spots are becoming more salient^2^

As gaps generated by IID evaluation procedures are becoming more salient, the search for understanding the reasons that cause these gaps is also gaining notoriety. The causative reasons go beyond the results presented in the training, they are related to the codified structure that is inherent to the model. When using ML, an important element is the prediction with good results in the training domain. However, in many contexts that use ML, for this to occur it is necessary that the ML application has learned through elements that make sense in the context in which it is inserted. For example, the requirements in response, where knowledge of the world is important, may differ from those in translation, where isolation of semantic knowledge is desirable^3^.

Researchers emphasize that contextual elements incorporated into the coded structure of ML models have practical consequences, as they profoundly impact whether the model can effectively generalize in real-world deployment scenarios. Thus, these elements serve as critical determinants of a predictor’s trustworthiness in practical use. Interestingly, even when two predictors are identically trained, they may exhibit different defects in production due to their ability or inability to learn from contextually relevant elements. Consequently, even predictors trained to the same level of generalization, when evaluated using the IID evaluation procedure, often display divergent behavior when applied to real-world settings. This divergence can be attributed to the existence of multiple solutions that address the problem in a similar manner. In the context of creating ML models for general problem-solving, a single algorithm can generate multiple solutions, achieving equivalent performance in IID evaluations through slight variations in feature weight settings or data patterns during training^3^. Thus, regardless of the application domain, a specific problem can result in the generation of numerous ML models using the same algorithm.

The reliance on IID evaluation procedure measures alone leads to the unreliability of ML models in practice. This limitation becomes critical when deploying models, as selecting a model based on a single evaluation metric prevents reporting on its robustness against naturally occurring changes in data distribution^4^. Consequently, ensuring model reliability has become a crucial aspect of modern ML, as it poses the main obstacle in determining whether a trained model will behave as expected in a production environment^3^.

In our work, the concept of reliability is closely tied to evaluating whether the training dataset environment adequately supports effective learning of a ML model. Specifically, we examine whether the models created from a given set of training data can maintain their performance during the deployment stage.

**Figure 1:**
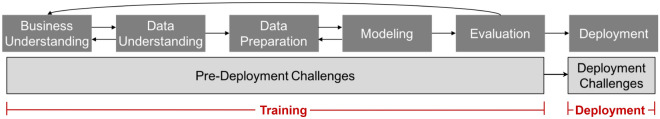
The two steps that span the lifecycle of a ML pipeline, training and deployment.

To provide a clearer understanding of our work’s context, we have developed Fig. 1 based on the research by Baier and colleagues^5^. This figure highlights two fundamental steps that constitute the ML pipeline’s cycle: the training stage and the deployment stage. The training stage addresses pre-deployment challenges, while the deployment stage focuses on real-life model usage. During the training stage, where the evaluation procedures follow the Independent and Identically Distributed (IID) principle, the model’s effectiveness becomes the primary concern. These evaluation methods solely measure the number of correct answers, overlooking the model’s learning capability. As a result, when the model transitions to the deployment stage, where it faces real-world data, it may exhibit unexpected behavior and yield results that significantly differ from expectations.

This uncertainty stemming from the limitations of IID evaluation procedures provided an opportunity for our work. To address this, we have developed a novel methodology that complements IID evaluation procedures, enabling a double-check to ensure that the created models are fully prepared for the production phase. Consequently, our approach enhances the overall reliability of model performance, particularly in handling naturally occurring changes in data distribution.

### Related works

Because problems in learning an ML model can cause undesirable results when deploying the model in the production environment, the question arises as to whether there is any way to resolve or mitigate this issue. Based on studies and research in the literature, two options were found that are used. The first option is through the use of A/B tests, in which, in the context of ML and the problem presented, it works by submitting the current model (control) that is in production to evaluation methods, together with the new model that was developed (variant). Therefore, the results of the A/B tests must be monitored for a certain time to provide statistical significance in defining the winner^6^. The second option is through the use of stress tests. Since the model has not been tested in the real world, the use of stress tests aims to run a series of tests to try to find gaps in the model’s learning and thus correct them before the model is put into production^7^.

Having presented the two options found in the literature and their main characteristics, we highlight the following points: Regarding the first option, we have the disadvantage of having as a prerequisite the need to have a previous model for its use, as the existing model needs to be used in comparison with the competing model to validate the results of the new model. Another disadvantage of this solution is the associated computational and time costs. Although there is no exact quantification for this, it is explicit in the literature that it is necessary to keep two models running in the same data configuration for a certain minimum period of time to achieve statistical significance.

The second option, which uses stress tests, does not require a model in production; however, as the name suggests, this solution requires a large number of tests to be carried out to stress the model in different directions. D’Amour et al.^3^ and collaborators cite several works^8–13^ in which the stress testing solution was the option used to treat cases in which the models did not generalize in the real world as predicted. As can be seen, there are a large number of stress-testing implementations; however, these tests can be divided into three types: stratified Performance Evaluations; shifted Performance Evaluations; and contrastive Evaluations. Below, we briefly describe each of these.

- *Stratified performance evaluations:* these types of tests perform stratified evaluations to see if a predictor encodes inductive biases that yield similar performance across different strata of a dataset.
- *Shifted performance evaluations:* test whether the average performance of a predictor generalizes when the test distribution differs in a specific way from the training distribution.
- *Contrastive evaluations:* contrastive tests support the localized analysis of particular inductive biases. Specifically, contrastive evaluations performed can verify whether a specific modification of the input causes the model’s output to change in unexpected ways.

As mentioned, the stress testing option is quite useful and is widely used in many jobs. However, as we can also observe, there is no pattern of stress tests to be followed; that is, there are several different sets of stress tests applied in the studies. Therefore, there is no standard method that can be applied to verify whether it is possible to trust that the model in question will work as expected in production. This alternative also has a high computational and time cost, owing to the exhaustive number of tests that are applied.

As stress tests are divided into three different types, because each test encompasses a series of complexities and specificities, in the present work, we chose to carry out an initial validation of our methodology only in the context in which the Shifted Performance Evaluations stress tests operate, which is a context in which there is a specific divergence in the distributions of the datasets. Once our methodology has been validated for this context, we will continue with a second study for the other two missing contexts. We chose to act in this context because we believe that among the three types of tests presented, this would be the one with the least modeling complexity to create an automated process at this first moment.

It is important to point out that although the focus of our methodology is on the specific context of one of the stress tests applied by D’Amour et al.^3^ in that first moment, our methodology does not act in the same way as this stress test. Although the stress test identifies specific characteristics of the datasets, our work identifies the problem generated from these specific characteristics in binary classification on tabular data, which would be an incomplete learning of the algorithms. In addition, to achieve our goal, instead of using defined rules and statistics, we created a methodology that has as a final result a ML model that will be used for this purpose.

### Item response theory

Once the related work has been presented, we will go into more detail on the methodology we have developed to help with the question of the reliability of the models for the production stage. The objective of this work is to provide an alternative that allows verifying that the ML model can be put into production through a pipeline that does not need to be adapted for each specific problem and is not dependent on a previously validated model.

To achieve this objective, we explored the Item Response Theory (IRT), which is a group of modeling and statistical tools designed to provide a precise characterization of items based on analysis of their responses^14^. According to Embretson and Reise^15^, the IRT is the psychometric basis for tests that have become the main theoretical basis for measurement; thus, important tests in the world use it to measure the performance of respondents, such as the Scholastic Aptitude Test (SAT), which aims to measure the level of knowledge of high school students in the United States^16^. This measurement theory aims to explore the ability of models and provide a better understanding of their learning process in relation to context.

In the context of ML, IRT can generate relevant information from hyperparameters for understanding the model and the model training context. The term “hyperparameters” refers to parameters that are not directly estimated from the observed data. These hyperparameters influence the behavior and properties of the IRT model. The specific hyperparameters can vary depending on the type of IRT model being used. In our work we utilized the 3-parameter logistic model, commonly referred to as 3PL^17^. This IRT model uses the following three hyperparameters:

- *Discrimination:* measures the item’s capacity to differentiate between highly and low-skilled respondents. The higher the discrimination value, the greater the item’s ability to separate a skilled from a less skilled respondent.
- *Difficult:* measures how difficult the item is to answer correctly. The higher the difficulty value, the more difficult the item is to answer correctly.
- *Guessing:* measures the chance that a low-skill respondent will hit an item by chance. The higher the high guessing value, the more likely a low-skilled respondent is to hit an item by chance.

To enable the classification of an ML context as unreliable, we leveraged the key characteristic of Item Response Theory (IRT), which allows us to evaluate respondents’ (in our case, ML models) performance directly on the items, considering the specific complexity of each item for more precise ability measurement.

In the evaluation process, the IRT utilizes the $\theta$ parameter to measure the respondent’s proficiency and calculate the probability of success. For a specific item, the probability of an individual *j* correctly responding to item *i* based on their ability is determined using the 3-parameter logistic model (3PL) of the IRT. This probability is defined by the following equation^17^:

1 $$\begin{aligned} P(U_{ij} = 1|\theta _{j}) = c_{i} + (1 - c_{i})\frac{1}{1+ e^{-a_{i}(\theta _{j}-b_{i})}} \end{aligned}$$

Where $U_ {ij}$ represents the dichotomous response, assuming values 1 or 0, where 1 indicates when individual *j* correctly answers item *i*, and 0 denotes a miss. $P(U_{ij} = 1|\theta _{j})$ stands for the probability of individual *j* providing the correct answer to item *i*. $b_{i}$ corresponds to the item’s difficulty hyperparameter, determining the position of the logistic curve, thereby indicating the level of difficulty for item *i*. $a_{i}$ represents the item’s discrimination hyperparameter, signifying how effectively item *i* distinguishes between respondents of different abilities. This hyperparameter governs the slope of the logistic curve, with higher values indicating greater discriminatory power. $c_{i}$ refers to the guessing hyperparameter, which signifies the probability of a casual hit. This parameter quantifies the likelihood that a respondent with low ability answers the item correctly.

Therefore, when using IRT in the context of ML pipelines, it is possible to obtain a different view from the usual one related to the evaluation of algorithms. The IRT provides a view that is not centered solely on IID evaluation, but seeks the true ability of algorithms^14^. The search for the true ability can be traced in different ways in the IRT because of the different ways in which it is implemented. However, most of these methods allow that discrimination, difficulty, and guessing hyperparameters to influence their results. Therefore, in this context, this study uses these three hyperparameters to build a methodology that makes it possible to identify whether the context in question is reliable for creating appropriate models.

Having discussed the significance of IRT and its potential contribution to solving the target problem, we aim to provide a clearer understanding of our work within the context of ML. For this purpose, we created a modified equation (Eq. 2) based on the learning illustration presented by Domingues et al.^18^.

2 $$\begin{aligned} Learning=Representation+Evaluation+Optimization+IRT \end{aligned}$$

In this equation, the “Representation” parameter refers to the space of permissible models, encompassing various model types such as decision trees or support vector machines with RBF kernels, each representing distinct modeling approaches. The “Evaluation” parameter plays a crucial role in distinguishing between good and bad classifiers, facilitating the assessment of model effectiveness. The “Optimization” parameter involves the process of searching the space of represented models to achieve improved evaluations, ensuring the selection of the most suitable model.

To enhance our methodology and complement the traditional learning components, we introduce a new parameter called “IRT,” which involves additional validation using hyperparameters not directly estimated from the observed data (such as difficulty, guessing, and discrimination). This inclusion empowers our approach with a more comprehensive model evaluation and verification, leading to greater reliability in real-world applications. By leveraging IRT, we gain deeper insights into model performance, making it more ready for deployment in production.

Additionally, to elucidate the relationship between IRT and the concept of reliability in our study, we constructed a hypothetical case that exemplifies the use of IRT in two ML scenarios: Scenario 1 and Scenario 2. Both scenarios involve binary classification problems with unbalanced datasets, where the majority class accounts for 80% and the minority class for 20% of the data. Following completion of all stages in the pipeline, we observed that both scenarios yielded identical accuracy rates of 80% and F1 Scores of 75%. However, when deployed in a production environment, only the Scenario 2 model managed to maintain results similar to those achieved during the training phase. In contrast, the Scenario 1 model exhibited a significant drop in performance, with an accuracy of 45% and an F1 Score of 40%.

After analyzing these hypothetical scenarios derived from actual observations, it becomes evident that relying solely on accuracy and F1 score results does not facilitate differentiation between the two scenarios. However, when applying our methodology based on IRT, it is possible to verify that the instances within the training dataset of Scenario 1 exhibited a specific combination of hyperparameters that, according to our ML model, suggests the instances within the training dataset were inadequate for effective learning of a model. Consequently, the accuracy and F1 score results achieved by the models trained on this dataset would likely not be sustained if deployed in a production environment. This was identified only in Scenario 1. Thus, in our study, we characterize the context of Scenario 1 as unreliable while considering the context of Scenario 2 as reliable.

It is essential to emphasize that, in this first moment, the concept of “unreliable contexts” in our work specifically refers to the context in which the Shifted Performance Evaluations stress tests operate (when there is a divergence in the distributions of the datasets) in binary classification using tabular data. Although our methodology aims to provide a complementary verification to determine if the model is ready for deployment, regardless of the context of the target problem, we initially chose to focus on this direction due to the abundance and variety of publicly available data with these characteristics.

## The proposed IRT based methodology

Once the negative points of the existing alternatives to deal with the problem of reliability in ML contexts and the contributions of the present work have been presented, we will provide more details about the study that allowed us to provide a solution to mitigate the problem in question and thus avoid the emergence of undesirable results when deploying the ML models into production.

**Figure 2:**
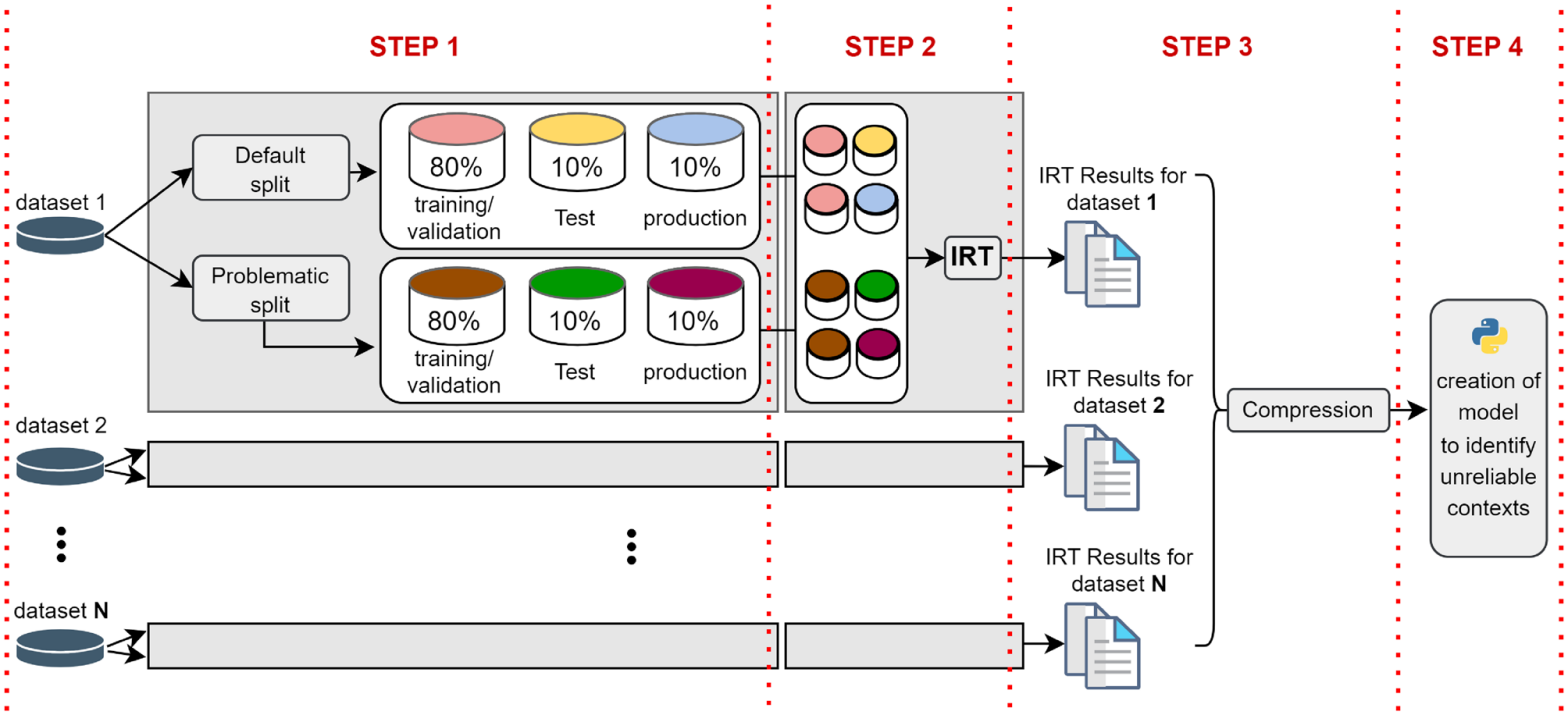
Methodology overview.

In this section, we will provide more detail about the methodology we have developed. However, to aid in understanding the methodology as a whole, we will first provide a general description and present a high-level overview that condenses its main processes (Fig. 2). Afterward, we will proceed to detail each of its processes.

The main objective of our work is to develop a model capable of identifying unreliable ML contexts related to the stress test of shifted performance evaluation. Our methodology, in a general description, is structured into four key steps. In Step 1, we utilize several tabular datasets of binary class and create two types of environments from each dataset: a normal environment and an unreliable environment (problematic environment). Each environment is further divided into three partitions: training/validation, testing, and production. The training/validation and testing datasets were created to adhere to the standard procedure in a machine learning pipeline. Thus, we utilize the training/validation set to train the model and keep a separate test set for evaluation. In addition to setting aside 10% of the data for testing, we also allocate another dataset of equal size to simulate the model’s real-world application (production dataset). The objective is to have two datasets of the same size that were not employed during the training phase. This allows us to evaluate the models’ ability to generalize to real-world data changes and determine whether the acquired knowledge is adequate or inadequate.

In Step 2 of our methodology, we arrange all the dataset partitions to be executed in pairs, resulting in four pairs for each dataset. These pairs include: the normal training partition with the normal test partition, the normal training partition with the normal production partition, the problematic training partition with the problematic test partition, and the problematic training partition with the problematic production partition. Subsequently, we apply the Item Response Theory (IRT) to extract the discrimination, difficulty, and guessing hyperparameters from each execution.

Moving on to Step 3, we condense the hyperparameter values obtained from each execution using both average and median techniques. This step is crucial due to the varying number of instances in the datasets. By adopting this approach, we ensure that datasets with more instances do not exert a disproportionate influence compared to datasets with fewer instances. The condensed values are then amalgamated into a single table for further analysis and examination.

Finally, in Step 4, we use this consolidated table as the foundation to carry out the entire ML model creation process. This process encompasses various critical components, including outlier removal, feature selection, splitting datasets with stratified cross-validation, model benchmarking, and ultimately selecting the most suitable and reliable model. By following this well-structured methodology, our work aims to provide a comprehensive approach for effectively identifying and evaluating unreliable ML contexts, leading to the development of more robust and reliable ML models.

Once explained the methodology that we created in a general way. We will continue below with subsections to explain each of the processes in depth. The processes will be described in subsections and will be detailed following the order in which they occur.

### Step 1: splits of dataset and creation of environments

As prerequisites of first step for creating our methodology, we only have that the datasets must have a binary class and that the number of datasets used as a base is greater than or equal to two. However, it is worth mentioning that since our objective is to try to improve the chances of our model generalizing, then the greater the number of datasets, the greater our possibilities of work and consequently the greater our probabilities of succeeding in our generalization.

The first step is performed for each dataset individually and is organized into two parallel processes, process 1 and process 2. Both processes divide the original dataset into three subsets: training/validation, testing, and production, following the split of 80% of the total instances for the training/validation set, 10% for the test set, and the remaining 10% for the production set. The difference between the two processes lies in their objectives. Process 1 aims to create a favorable environment for the ML context, while process 2 intentionally forces the creation of a problematic environment (unreliable environment). In process 1, the three datasets are generated in such a way that they exhibit a very similar distribution of feature values and class proportions. Conversely, process 2 is designed to enforce the creation of a problematic environment related to the context of shifted performance evaluations. This involves intentionally introducing small divergences in data distribution among the training/validation, test, and production environments.

**Figure 3:**
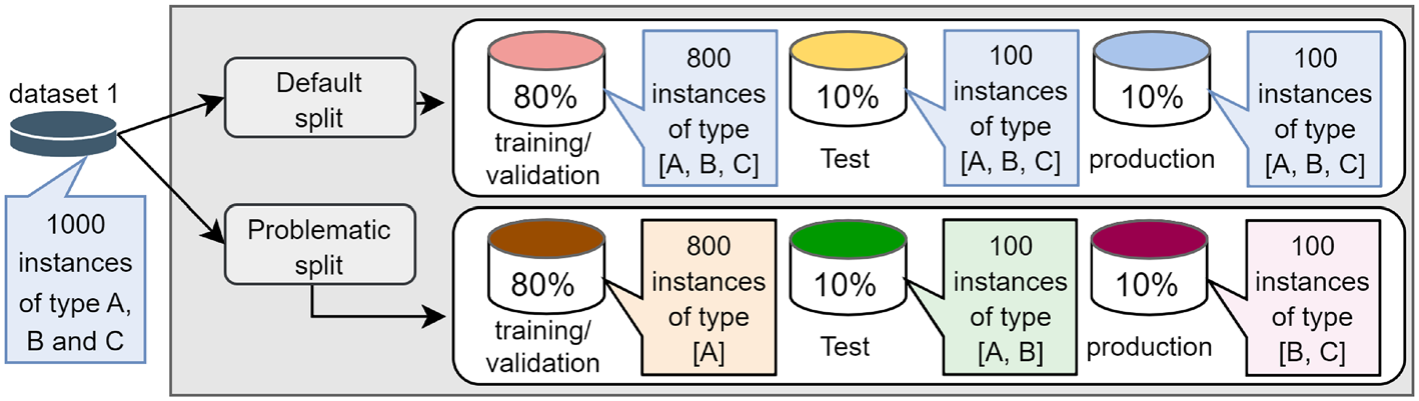
Example of creating the normal and unreliable environment from a dataset with 1000 instances.

As explained, in Step 1 each one of the datasets goes through two splitting processes. Since the same data instances that exist in the unreliable environment also exist in the normal environment, the difference between the environments is the group of instances that will be contained in each of the partitions that were created, which will consequently influence the learning of the algorithms. In Fig. 3, we present an illustrative example showcasing the processes involved in Step 1 for a base dataset comprising 1000 instances. The base dataset, denoted as dataset 1, contains instances with diverse characteristics represented as types A, B, and C, each signifying distinct attributes within the dataset.

For the default split, our aim is to maintain a similar distribution of the dataset, ensuring all types of instance characteristics are present in each partition to facilitate complete learning. To achieve this, we applied Stratified Sampling ten times and subsequently used the Kolmogorov-Smirnov test to select the execution of Stratified Sampling that resulted in partitions with the highest similarity between them (training/validation, test, and production).

On the other hand, in the problematic split (implemented to create the unreliable environments), we deliberately induced a divergence in the distribution between the partitions. As a result, some partitions lack certain data characteristics. For instance, in Fig. 3, we observe that the problematic split produced a training/validation partition without any instances of type B and C. Consequently, the model will not be able to learn the characteristics associated with these instance types.

Until now, we have not found in the literature steps defined related to problematic environments related to the stress test of shifted performance evaluation. As a result, our process was developed based on relevant studies and research in the field. It is important to note that our intention is to construct environments that challenge the algorithm’s learning process, driven by the specific characteristics of a given ML context, rather than adhering to a fixed set of predetermined rules. In other words, these specificities may manifest in diverse ways, as our focus is not on any particular predefined structure but rather on factors influencing the algorithm’s learning behavior. Consequently, our primary emphasis in process 2 (which generates an unreliable environment) is to create an adverse setting that deprives the algorithm of essential information necessary for comprehensive learning. By doing so, we can thoroughly evaluate the algorithm’s performance under real-world challenges, enhancing the robustness and reliability of our approach.

Thus, to create the respective unreliable environments for each dataset, we follow a series of steps. First, we perform a correlation analysis to identify the feature (referred to as Feature X) that exhibits the strongest correlation, either positive or negative, with the target variable. Once Feature X is identified, we rearrange the dataset in ascending order based on its values. With the reordered dataset, we divide it into three distinct sets: the training/validation dataset comprises instances with the lowest values in Feature X, accounting for 80% of the total instances. The remaining instances are divided into two blocks, with one block forming the test dataset (10% of the total instances) and containing instances with the lowest Feature X values, while the other block forms the production dataset (10% of the total instances).

It is important to note that the split employed to create the unreliable environment does not adhere to best practices in ML and is not utilized in real-world applications due to its potential to introduce learning problems. However, for our specific experimentation purpose of inducing incorrect learning, this process becomes appropriate.

Upon completion of the two processes within Step 1, we generate a total of six datasets for each base dataset: three datasets representing the normal environment (training/validation, test, and production) and three datasets representing the unreliable environment (unreliable training/validation, unreliable test, and unreliable production).

### Step 2: organization for DecodIRT and execution

In Step 2 of our methodology, all generated datasets are organized in pairs and executed in DecodIRT. DecodIRT is a tool that implements the IRT using the 3PL logistic model^19^. As mentioned in the previous section, the 3PL logistic model was used because it uses three item hyperparameters that have important information for the evaluation of the complexity of the datasets and for the calculation of the ability estimate of ML models. The DecodIRT tool was developed in Python and can be run via the command line. Therefore, we adapted the tool and incorporated it into the code of our methodology.

To calculate the IRT estimators, our methodology generates several models using Scikit-Learn^20^. By default, our tool estimates the ability and hit probability using 19 different classifiers, of which 12 are real classifiers and 7 are artificial classifiers. The 12 real classifiers are: Naive Bayes Gaussian, Naive Bayes Bernoulli, 2-neighbor KNN, 3-neighbor KNN, 5-neighbor KNN, 8 neighbors, Decision Trees, Random Forest (RF) with 3 trees, Random Forest with 5 trees, Random Forests, SVM and MLP. The selection of these classical models is guided by the research conducted by Cardoso and their collaborators^19^. Their primary objective was to incorporate diverse algorithmic families to cover various proficiency levels and characteristics without introducing excessive complexity into the models, thereby enhancing the diversity of responses generated by the classifiers without incurring excessive processing costs. The 7 artificial classifiers were proposed by researchers^14^ to be used as a baseline for the evaluation of the other models.

All datasets are organized in pairs for execution in DecodIRT because each execution requires two datasets as input parameters: Input 1 and Input 2. The dataset of Input 1 is utilized to train the models, while the dataset of Input 2 is used for evaluation and to generate estimates related to the IRT. In our methodology, among the various results produced by DecodIRT, we specifically utilize the results related to the estimated values of the discrimination, difficulty, and guessing hyperparameters for each instance in the Input 2 dataset. Consequently, after each execution, we obtain a table as the result. For instance, if the dataset partition inserted in Input 2 contains 1000 instances, the outcome of DecodIRT will be a table with 1000 rows and 4 columns—one column for the instance ID and one column for each hyperparameter (discrimination, guessing, and difficulty).

To better illustrate the pairwise organization, we have created Table 1, which demonstrates the partitioning process for a single dataset. Considering that each dataset is converted into six dataset partitions in Step 1, Table 1 shows that out of the six existing partitions, four runs are performed in DecodIRT—two for each environment (normal and unreliable). Specifically, two executions occur for the normal environment using different subsets of the data (test dataset and production dataset in Input 2), and the same applies to the unreliable environment.

**Table 1:** Example of organizing run rounds for partitions generated by a single dataset for IRT.

Input of dataset 1	Input of dataset 2	Dataset environment type
Training/validation	Test	Normal
Training/validation	Production	Normal
Training/validation	Test	Unreliable
Training/validation	Production	Unreliable

The purpose of organizing the datasets in this structure was to enable the evaluation of two different types of comparisons. The first comparison is intended to assess the differences between the results of the test and production environments. The second comparison aimed to evaluate the difference between the results of normal environments and those of unreliable environments. Therefore, once all execution runs for all datasets are structured, DecodIRT is executed for each run.

### Step 3: compression of results

An important observation in our methodology is that we utilize the estimated values of the discrimination, difficulty, and guessing hyperparameters for each instance in the Input 2 dataset. As a result, the number of instances present in the result of each round varies based on the number of existing instances in Input dataset 2. Consequently, the results generated from each execution of DecodIRT need to undergo a comprehension process due to the varying instance counts for each result.

To ensure equal treatment of all datasets (without giving more weight to datasets with larger instances, for example), in Step 3, we calculate the median and mean values of all hyperparameters (discrimination, difficulty, and guessing) for each round of results. In Table 2, we provide an example of how the compression of the results would appear if there were only two datasets in the base of the methodology.

**Table 2:** Example of the compression of results if there were only two datasets in the methodology base.

Input 2	Env	Mean-diff	Median-diff	Mean-guess	Median-guess	Mean-disc	Median-disc
dataset_1_test	Normal	− 1.12	− 0.20	1.62	1.42	− 1.32	− 0.58
dataset_1_prod	Normal	− 2.27	− 2.23	2.02	1.58	− 0.98	− 0.87
dataset_1_test	Unreliable	1.44	1.36	1.84	1.77	1.53	1.32
dataset_1_prod	Unreliable	2.18	2.12	1.12	0.12	3.02	2.70
dataset_2_test	Normal	− 1.14	− 0.71	2.32	2.12	1.12	0.58
dataset_2_prod	Normal	− 1.42	− 1.20	0.92	0.72	− 2.82	− 2.58
dataset_2_test	Unreliable	1.84	1.21	1.22	1.16	1.72	1.48
dataset_2_prod	Unreliable	1.67	1.43	2.02	1.58	1.80	1.47

Abbreviations present in the table: prod for production; env to environment; diff for difficult; guess for guessing; disc for discrimination; under for unreliable.

### Step 4: machine learning model to classify unreliable environments

In Step 4, our objective is to create an ML model capable of classifying normal and unreliable environments using the clustered and condensed results obtained in Step 3. To achieve this, we followed common steps for building an ML model. First, we explored the dataset to identify potential filters or treatments that might be necessary. The base dataset maintains the structure presented in Table 2, comprising only six features: mean difficulty, mean guessing, mean discrimination, median difficulty, median guessing, and median discrimination. The “env” column represents the target variable for classification.

During the Application of Filters and Treatments stage, we applied necessary treatments, filters, and removed outliers as required to prepare the data. For the Training phase, we performed a split using stratified cross-validation with 10 folds, allocating 80% of the data for training and 20% for testing.

From the generated splits, we proceeded to create several ML models, including GaussianNB, BernoulliNB, lightGBM, XGBoosting, AdaBoosting, Random Forest, and Gradient Boosting. Finally, in the Models Evaluation and Selection step, we thoroughly evaluated the performance of each model, choosing the one that demonstrated the best results.

As the last step of our methodology is Step 4, from the conclusion of this step, we have an ML model capable of classifying normal and unreliable environments (unreliable environments related to the context of the Shifted Performance Evaluation).

## Results

Once we describe our methodology, this section presents the data and results used to create our classification model for normal and unreliable environments. For our main database, we used 21 binary-class datasets obtained using OpenML. OpenML is a collaborative platform that allows users to share data, experiments and algorithms^21^. In Table 3 we can see the identification of all datasets used and the information related to the features and dimensions of each of them.

**Table 3:** The table presents the identification of the datasets used by OpenML and the information related to the features and dimensions for each of them.

Dataset identification	Instances × features	Categorical x numerical (features)	Class balancing
Banknote-authentication	1372 × 5	0 × 5	55–45%
Bioresponse	3751 × 1777	0 × 1777	55–45%
Blood-transfusion-service-center	748 × 5	0 × 5	76–24%
Breast-w	683 × 10	0 × 10	65–35%
Climate-model-simulation-crashes	540 × 21	0 × 21	99–1%
Cylinder-bands	277 × 40	15 × − 25	64–36%
Dresses-sales	99 × 13	11 × 2	0.59–0.41%
Diabetes	768 × 9	0 × 9	65–35%
ilpd	583 × 11	1 × 10	71–29%
Internet-advertisements	3279 × 1559	0 × 1559	0.87–0.13%
kc1	2109 × 22	0 × 22	0.84–0.16%
kc2	522 × 22	0 × 22	0.79–0.21%
Mandelon	2600 × 501	0 × 501	0.5–0.5%
Ozone-level-8hr	2534 × 73	0 × 73	0.93–0.07%
pc1	1109 × 22	0 × 22	0.93–0.07%
pc3	1563 × 38	0 × 38	0.89–0.11%
Phoneme	5404 × 6	0 × 6	0.70–0.30%
qsar-biodeg	1055 × 42	0 × 42	0.66–0.34%
Spambase	4601 × 58	0 × 58	0.60–0.40%
wdbc	569 × 31	0 × 31	0.62–0.38%
wilt	4839 × 6	0 × 6	0.94–0.06%

The 21 datasets were then sent to Step 1 of the methodology, which is the step responsible for splitting the dataset and creating the environments. As a result of this step, each dataset generated six datasets, since they are two different environments, and for each environment we have a training/validation dataset, a test dataset and a production dataset. After completing this first step, we obtained 126 datasets.

The 126 datasets were sent to the step that organizes the datasets in pairs and executes them in DecodIRT. In this step, the 126 datasets were organized to be used in 84 rounds of execution in DecodIRT (following the structure presented in the Table 1 for each dataset). Soon after the organization, the rounds were executed.

After all the executions, we conducted a check to confirm whether the normal and unreliable environments presented themselves in different ways. For this, we performed an analysis to compare the differences between the results obtained in the test and production datasets for each environment. To perform this analysis, for each execution, we extracted the accuracy results for each of the algorithms obtained in the production environment and subtracted them from the results obtained in the test environment. Subsequently, absolute values of the differences were calculated.

**Figure 4:**
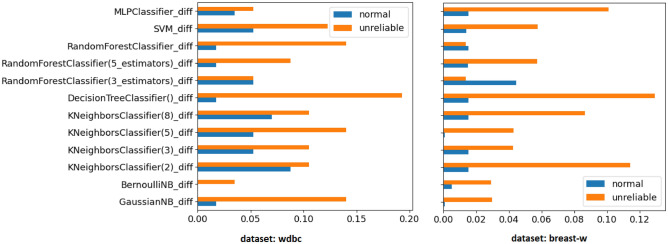
Differences in accuracy values obtained between test and production datasets for normal and unreliable environments for wdbc and breast-w datasets.

After completion of the comparative analysis, it was possible to confirm that the environments are different and that we managed to achieve our goal of creating an environment not suitable for the algorithms (unreliable environment). To visually demonstrate our confirmation, we present in Fig. 4 the absolute values of the differences between the accuracies obtained in the test and production partitions for each algorithm in the two environments (normal and unreliable) of the two datasets (wdbc and breast-w). In the Fig. 4, it can be observed that the absolute difference between the test and production accuracy values of the algorithms in an unreliable environment (orange bars) is much higher than the absolute differences presented in the normal environment (blue bars). Furthermore, we can also observe that of the 24 comparisons presented in the Fig. 4, only in one of them the difference presented in the normal environment is greater than the difference presented by a unreliable environment, and even in this case, the difference value is smaller compared with situations where the unreliable environment has a higher difference value. The figure presents only the results of two of our datasets, but this pattern extends to the other datasets.

The IRT results generated in Step 2 were then sent to the comprehension of the results (Step 3). In this step, statistics were applied to compress the hyperparameter values of the datasets. The final result of these applications follows the structure that was presented in the Table 2, and the complete table of results can be found in our repository.

The table of compressed results arrives at step 4, step responsible for creating the ML model to classify normal and unreliable environments. The compressed table with the results of the hyperparameters generated in step 3 is used in this step as a database for the construction pipeline of the classification model. Therefore, the first pass of the pipeline performed exploration and data processing. With the results of exploration, no treatment needed to be performed. After these steps, we train the selected models with a stratified cross-validation divided into 10 folders (80% for training and 20% for testing). The result of the evaluation step of the algorithms in the test dataset can be seen in the Table 4. As can be seen, in addition to using the accuracy and F1 score metrics, we also use the metric created by our methodology to assess whether normal or unreliable. As no context was classified as unreliable, the Gradient Boosting algorithm was chosen for presenting the best results in terms of accuracy and F1 score metrics.

**Table 4:** Accuracy and F1 score results with stratified cross-validation in the context of classification of unreliable environments.

ML algorithm	Accuracy	F1 score
GaussianNB	0.877	0.878
BernoulliNB	0.863	0.856
lightGBM	0.885	0.880
XGBoosting	0.891	0.889
AdaBoosting	0.882	0.876
Random forest	0.921	0.916
Gradient boosting	0.935	0.930

## Discussion

Our work demonstrated that, using the hyperparameters of difficulty, guessing, and discrimination, it is possible to determine whether a context is favorable or not (unreliable context) for learning of ML algorithms. That is, if the context is classified as unreliable by our ML model, it can be said that the trained algorithm has a high probability of not having learned properly, and this will cause the results obtained during model training to have a high probability of not being maintained in the deployment environment.

Therefore, we can conclude from this study that we were able to identify if a context is unreliable through a ML model (by now considering the issues related to the context of Shifted Performance Evaluation). Our algorithm achieved an accuracy of 0.935 and an F1 score of 0.930 in its final results. These values from a ML perspective indicate excellent results if the context is reliable.

In addition to the positive results related to the ML perspective, there are currently no pipelines in the literature that identify that an environment is unreliable without the use of rules that look for specific characteristics. The pursuit of these characteristics presents a drawback as it necessitates adaptation for each type of target context. Since our methodology is not rule-based, we utilized various datasets with varying characteristics and levels of complexity to introduce diverse challenges and behaviors in the environments. This approach allowed us to generate a variety of inputs for our model, enabling it to learn and generalize to other contexts effectively.

A limitation of this work is that, for this first study, we used only the context in which the tests of the Shifted Performance Evaluation were inserted. Although just one type of stress testing context is already a contribution to the scientific community and the fact that we are not using any specific rules suggests that our methodology will work in other contexts, we would also like to apply our methodology in other contexts to confirm our beliefs. Therefore, one direction that we will explore in future work is to conduct tests and analyses in other problematic contexts.

## Data Availability

This study obtained research data from publicly available online repositories. We mentioned their sources using proper citations. Here is the link to the data in our repository https://github.com/vitorcirilo3/reliability-identifier/tree/main/datasets.

## Code availability

All source code and guidelines are publicly available on https://github.com/vitorcirilo3/reliability-identifier.

## References

[1] Darrell T, Kloft M, Pontil M, Rätsch G, Rodner E (2015). Machine learning with interdependent and non-identically distributed data. Dagstuhl Rep..

[2] Geirhos R (2020). Shortcut learning in deep neural networks. Nat. Mach. Intell..

[3] D’Amour A (2020). Underspecification presents challenges for credibility in modern machine learning. J. Mach. Learn. Res..

[4] Ortiz-Jiménez, G., Salazar-Reque, I. F., Modas, A., Moosavi-Dezfooli, S. & Frossard, P. A neural anisotropic view of underspecification in deep learning. *CoRR* (2021). arXiv:2104.14372.

[5] Baier, L., Jöhren, F. & Seebacher, S. Challenges in the deployment and operation of machine learning in practice. In *ECIS 2019 proceedings . 27th European Conference on Information Systems (ECIS), Stockholm & Uppsala, Sweden, June 8–14, 2019. Research Papers*, Paper: 163 (AIS eLibrary (AISeL), 2019).

[6] Kaukanen, M. *Evaluating the impacts of machine learning to the future of A/B testing*. Master’s thesis, School of Engineering Science, Industrial Engineering and Management (2020).

[7] Young AT (2021). Stress testing reveals gaps in clinic readiness of image-based diagnostic artificial intelligence models. NPJ Digit. Med..

[8] Collins GS, Reitsma JB, Altman DG, Moons K (2015). Transparent reporting of a multivariable prediction model for individual prognosis or diagnosis (TRIPOD): The TRIPOD statement. BMC Med..

[9] Liu X (2020). Reporting guidelines for clinical trial reports for interventions involving artificial intelligence: The CONSORT-AI extension. Nat. Med..

[10] Rivera SC (2020). Guidelines for clinical trial protocols for interventions involving artificial intelligence: The SPIRIT-AI extension. Lancet Digit. Health.

[11] Mullainathan S, Spiess J (2017). Machine learning: An applied econometric approach. J. Econ. Perspect..

[12] Athey S (2017). Beyond prediction: Using big data for policy problems. Science.

[13] Kleinberg J, Ludwig J, Mullainathan S, Obermeyer Z (2015). Prediction policy problems. Am. Econ. Rev..

[14] Martinez Plumed F, Prudenco RBC, Martinez Uso A, Orallo JH (2016). Making sense of item response theory in machine learning. Front. Artif. Intell. Appl..

[15] Embretson, S. E. & Reise, S. P. *Item Response Theory for Psychologists (Multivariate Applications Series)* (Psychology Press, 2000).

[16] An X, Yung Y-F (2014). Item response theory: What it is and how you can use the irt procedure to apply it. SAS Inst. Inc..

[17] Baker, F. B. *The Basics of Item Response Theory* (ERIC, 2001). 10.1177/0146621617748327 PMC6023097 30034058

[18] Domingos P (2012). A few useful things to know about machine learning. Commun. ACM.

[19] Cardoso, L. F. F., Santos, V. C. A., Francês, R. S. K., Prudêncio, R. B. C. & Alves, R. C. O. Decoding machine learning benchmarks. In *Intelligent Systems* 412–425 (Springer International Publishing, 2020). 10.1007/978-3-030-61380-8_28.

[20] Pedregosa F (2011). Scikit-learn: Machine learning in python. J Mach. Learn. Res..

[21] Vanschoren J, van Rijn JN, Bischl B, Torgo L (2014). OpenML. ACM SIGKDD Explor. Newsl..

